# The Other “A” Word: Assessing the Accessibility of Abortion in Academia

**DOI:** 10.1089/whr.2021.0066

**Published:** 2022-02-02

**Authors:** Sarah M. Jordon, Ian S. Ray

**Affiliations:** ^1^ Department of Higher Education, University of Denver, Denver, Colorado, USA.; ^2^ Dr3 Research and Consulting, LLC, Denver, Colorado, USA.; ^3^ Department of Research Methods and Information Science, University of Denver, Denver, Colorado, USA.; ^4^ Department of Anthropology and Sociology, Red Rocks Community College, Lakewood, Colorado, USA.; ^5^ Department of Social Sciences, Community College of Aurora, Aurora, Colorado, USA.

**Keywords:** women's health, colleges, abortion access

## Abstract

***Objective:*** To assess the accessibility of abortion providers across collegiate campuses in the State of Colorado.

***Participants:*** Analysis was on secondary data from the Integrated Postsecondary Data System and the U.S. Census Bureau.

***Methods:*** We utilized a framework of Reproductive Justice to assess and interpret the accessibility of abortion providers to college students by mapping reproductive health centers and nonprofit institutions of higher education, then using these data to statistically compare racial demographics, female employment, and insurance coverage between institutions with and without access.

***Results:*** Of nonprofit postsecondary institutions in Colorado, 11 institutions lack access, serving ∼38,900 students. Of these students, 88.7% attend a Minority Serving Institution (MSI). MSIs lacking abortion access had 8% more female enrollment [*t*(18.32) = −2.04, *p* = 0.027]. Campuses possessing student health centers are less likely to have an MSI designation (50% with vs. 82% without), have greater female Hispanic enrollment by 10% [*t*(23.72) = 3.11, *p* = 0.005] and lower female multiracial enrollment by 2% [*t*(37.00) = 2.20, *p* = 0.034]. Analysis of variance (ANOVA) results indicated significant differences in percent composition of Asian, black or African American, Two or More, and Nonresident demographics between collegiate campuses with 30-, 45-, and 60-minute drive-time access. ANOVA results indicated separate patterns of differences for Zip Code Tabulation Area (ZCTA) demographics for Asian, black or African American, Native Hawaiian or Pacific Islander, Other Race, and Nonresident populations. Planned contrasts demonstrated that this difference was greatest at the 30-minute drive-time access, supporting the use of 30-minute drive time as an important indicator of access.

***Conclusions:*** The colleges lacking access to a provider are predominantly MSIs in lower income communities. The patterns in campus and ZCTA demographics indicate that collegiate populations are affected by rurality differently than the general population. We recommend that future qualitative research to assess rural students' perceptions on access, campus health center practices, and practices on campuses without dedicated health facilities.

## Lay Language Statement

We argue that the current distribution of reproductive health care facilities offering voluntary fetal termination disproportionately disadvantages Minority Serving Institutions and surrounding communities.

## Introduction

The systematic interactions between health care, student services, and accessibility is a key component of understanding the student experience of higher education, particularly for individuals who find themselves with an unplanned pregnancy. Although we were unable to find recent data on the utilization of abortion services by college students, an estimated 650,000 known abortions being performed annually across the United States, with ∼58.9% of those receiving this service being women aged 20–29, which is within traditional college-going age range.^1^ Young women aged 15–19 account for an additional 10.4% of known abortions performed.^1^

In this article, we specifically focus on college students' physical access to abortion clinics in Colorado. Proximity to abortion clinics is a pertinent part of the abortion conversation, as distance impacts access and many individuals may not have the means to seek a safe abortion if the travel burden associated with accessing one is too high and/or costly.^2^ In Colorado, the urban and suburban areas, largely in the Front Range, include some of the fastest growing counties in the nation.^3^ As of 2016, 47 of 64 counties in the state are classified as either rural or frontier (6 or fewer persons per square mile) with an additional six (6) urban counties bordering one or more frontier counties.^4^

Colorado is also one of only 14 Democratic trifectas, with the state house, senate, and Governor's office all currently controlled by the party.^5^ This is noteworthy as access to safe and legal abortion was part of the 2016 Democratic Party Platform and a part of Democratic talking points for the 2020 elections.^6^ To make the results of this study actionable for policy-makers, we focused on comparing collegiate populations with the general public, with the intent of specific demographics for whom abortion access differentially impacts.

## Theoretical Framework

Articles focusing on college student's health in health-related journals revealed a lack of the utilization of social theory to explain and understand phenomena occurring across higher education institutions. Davies et al.^7^ found that a theoretical rationale was used in less than 10% of all studies focused on medical intervention. Although the study is over 16 years old,^7^ our subsequent review of the literature revealed that the utilization of a theoretical rationale remains underutilized in academic publications on college health and medical intervention. When theory is utilized, it is usually done in a way that guides analysis rather than being folded into the research design.^8^ We utilize intersectionality and reproductive justice to frame our research design in looking at abortion access in Colorado.

Intersectionality as a mode of analysis has become an interdisciplinary phenomenon,^9^ however, has yet to gain traction in health care research. Crenshaw^10^ first developed the term intersectionality as a legal framework to look at the lived experiences of women of color to highlight how they were often left out of political conversations. For example, there was recognition of (white) women, or (black) men, yet, conversations of black women were not happening.^11^ The same systematic issues and failure to recognize intersectionality has since become a contentious topic across the country with coronavirus disease 2019 (COVID-19) highlighting systematic health care inequities.

The utilization of intersectionality as a theoretical framework does not require all identities to be addressed in every analysis. Instead, the effectiveness of intersectionality requires a pointed focus on specific identities.^9^ To remain consistent with the utilization of an intersectional framework,^9^ we specifically focus on the intersections of socioeconomic status, rurality, race, and gender. These identities were chosen as a focus because of Colorado's concentrated wealth, vast rural areas, increasing minority, specifically Hispanic, populations, and the gendered nature of social impact in the abortion debate.

To further focus intersectionality on the topic of reproductive choice access, specifically abortion, we further narrow intersectionality down to Reproductive Justice. Reproductive Justice also began as a legal framework to understand marginalized women's systematic interactions with the legal system.^12^ As a framework, Reproductive Justice was created by women of color to organize communities in an effort to challenge structural inequalities and power when it comes to women's rights, specifically related to the rights of (1) having a child, (2) choosing not to have a child, and (3) raising their children.^14^

When utilizing Reproductive Justice to understand reproductive oppression, many argue that the framework of intersectionality is required, as it allows us to look at the totality of individuals' experiences.^14,15^ This totality encompasses not only individuals' motivations and actions but also the multilayered social environment that influences both understandings of reproductive care and access to facilities providing that care.^12^ We subscribe to Ross's^13^ understanding of Reproductive Justice as a framework, which is defined as:

The belief that systemic inequality has always shaped people's decision making around childbearing and parenting, particularly vulnerable women. Institutional forces such as racism, sexism, colonialism, and poverty influence people's individual freedoms in societies. Other factors—such as immigration status, ability, gender identity, carceral status, sexual orientation, and age—can also affect whether people get appropriate care.^13(p. 291)^

This definition allows us to move away from the essentialization of woman as an identity category when discussing abortion and instead look at a multitude of racial and socioeconomic factors that play into family planning access for our most vulnerable students. We used intersectionality and Reproductive Justice throughout to determine research questions, definitions of accessibility, and variables mapped throughout our analysis. This article expands on the conversation of abortion access by utilizing Geographic Information Science (GIS) software to visually overlay multiple risk factors to estimate reproductive choice access for college students across Colorado.

### Review of the literature

There is a dearth of literature available on the topic of reproductive choice as it relates to higher education, with the majority of current studies focusing on emergency contraception,^15^ birth control,^16,17^ and student perceptions of pregnancy and choice.^18–20^ Although all these studies contribute to the conversation of reproductive choice for higher education students, a topic drastically understudied, it is essential to center abortion in this research.

Pregnancy significantly increases the likelihood that a woman^1^ will drop out of school, although is not considered a significant factor for college drop-out rates overall.^21^ Women who give birth while attending community college are 65% less likely to finish their degree compared to their peers who do not have a child during that same time period.^22^ The outcomes for women who find themselves pregnant at a 4-year institution are no better.

Six out of eight women who find themselves pregnant during school will end up taking an incomplete in at least one of their courses, if not drop out of school entirely.^16^ We were unable to find any current statistics on what this number looks like across the United States nor anything disaggregating this number to account for the intersectionality of experience and factors, which could influence these numbers.

College-aged women, those between 18 and 29, account for 68% of unplanned pregnancies nationwide.^23^ This indicates a significant risk factor in women's access to and retention within higher education. An estimated $10 billion annually is spent in the United States alone, on direct medical costs and increased public assistance expenditures from pregnancy and childbirth in those younger than 20 years of age alone.^24^ Despite these statistics, campuses across the country provide limited, if any, information to students on pregnancy prevention and the choices available to students with an unwanted pregnancy.^15^

Nationally, only 38% of women in the United States live in a county with a known abortion provider.^15^ Colorado represents an anomaly with 88% of women living in such a county, yet, this may be due to the aforementioned growth along the Front Range region and not due to more uniform abortion access.^25^ Despite this, Colorado ranks 21st nationally and with only a C+ ranking on reproductive rights by the Institute for Women's Policy Research.^25^ To calculate this ranking, the Institute for Women's Policy Research^25^ looks at an array of data, including but not limited to, access to abortion, family planning policies and resources, and the Affordable Care Act and contraceptive coverage.

We found no other recent research looking at reproductive choice access among college students in Colorado, despite the state's 32 public 2- and 4-year institutions with a combined total of over 98,000 female-identifying students.^26^ The largest percentage of female students are younger than the age of 25 years, well within the statistical range of women most likely to have an unplanned pregnancy.^26^

Based on a preliminary analysis of subsequent research, we found that Colorado collegiate health centers may provide no or limited information to students on postconception reproductive choices. In addition, few 2-year institutions offer health centers on campus, further reducing access to all health care for students.

## Methods

All data were obtained from publicly available sources and without any individually identifiability information. Therefore, the University of Denver's IRB determined this study was not be human subjects research and is thus exempt from IRB review. These data included abortion provider locations from their respective websites, campus locations, and demographics from the Integrated Postsecondary Education Data System (IPEDS),^26^ and Zip Code Tabulation Area (ZCTA) shapefiles from the U.S. Census Bureau.^27^ Provider locations were mapped following standard geospatial techniques^28^ and all campuses within a 30-minute drive time of providers were removed from analyses.

Individual provider locations were intentionally masked to prevent the use of this research by terrorist activities, including “Abortion Bombing.”^29^ The potential of this happening was evidenced on August 20, 2019 when a man in Chicago was arrested after making threats to “slaughter and murder any doctor, patient, or visitor” at a women's health clinic located approximately four miles from his home.^30^

Previous studies have used varying distances as approximating access, with up to 100 miles termed accessible in some analyses.^31^ However, these studies failed to develop a unified geographic definition of access.^31–33^ Fuentes and Jerman^34^ found that 65% of patients traveled less than 25 miles to access a provider, while only 18% of patients traveled more than 50 miles. Our theoretical framework causes us to question if marginalized populations have access to sufficient resources required to travel such distances. In addition, Colorado's geographic landscape presents unique challenges to this analysis.

We estimate that a 30-minute drive time corresponds to a reasonable definition of abortion access. However, due to the potential for substantial variation in access to reliable transportation and perceived reasonable distances, we continued our analysis with 45- and 60-minute drive time estimates as well.

IPEDS-derived data indicate that the 39 nonprofit colleges and universities in Colorado enroll ∼321,100 students. Of these, ∼19.9% (63,900) attend a Hispanic- serving institution (HSI), defined as an institution that is at least 25% Hispanic enrolling, while 40.3% (129,700) attend an emerging Hispanic-serving institution (eHSI), which is defined as an institution enrolling at least 15% Hispanic students, but not yet 25%.^26,35^ Overall, 193,600 students (60.3%) attend a Minority Serving Institution (MSI) in Colorado.^26,35^

Our theoretical framework led us to a specific interest in female employment, uninsured populations, and Hispanic populations. These data were combined with data each assessing percent of unemployment, Native American, black, Asian, Pacific Islander, and Two or More racial categories to create an overall database for ZCTA in Colorado. Intersectional theory also leads us to question the impact of citizenship status, and while we include this in our overall assessment of risk, we do not provide a separate assessment due to the increasing frequency of “Abortion Bombing” terrorism^29^ as well as targeting of populations based on perceived immigrant status, evidenced by the recent attack in El Paso, TX, and anti-immigration discourse happening across the nation.

We utilized Student's *t*-tests to compare these demographic data between institutions with and without access as well as between ZCTAs with and without access. We then analyzed the publicly available data from the American Community Survey (ACS) to determine if there were any statistically significant differences in female employment or overall health insurance coverage between ZCTAs with and without abortion access. Because definitions of accessible reproductive health care differ, we utilized analysis of variance (ANOVA) with planned contrasts to compare institutions or ZCTAs with access in under 30-minutes drive time, 30- to 45-minutes drive time, 45- to 60-minutes drive time, and no access in under 60-minutes drive time.

## Results

**FIG. 1.**
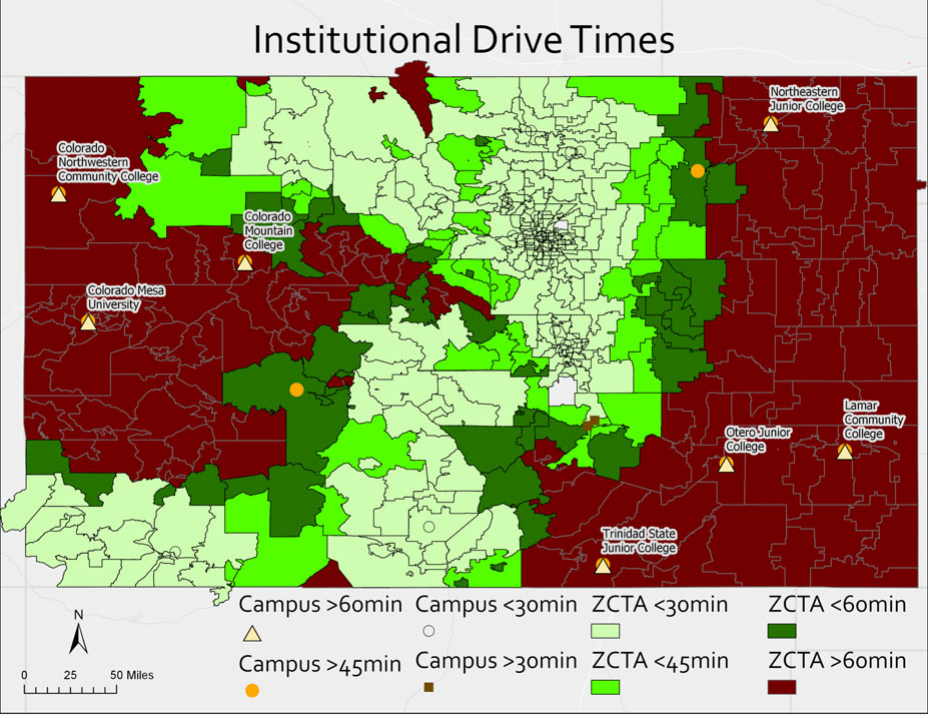
Institutional and ZCTA drive times. ZCTA, Zip Code Tabulation Area.

Of the 39 total nonprofit institutions, 28 fall within our definition of access, accounting for ∼282,200 students, or 87.9% of Coloradan students (Table 1; Figure 1). Of these, ∼46,100 students (5.5%) attend an HSI and 112,000 students (39.6%) attend an eHSI. Overall, 158,100 students (56%) attend a MSI within 30 minutes of an abortion provider.

**Table 1.** Institutions with Access

**Institution**	**Total enrollment** ^27^	**Hispanic-serving status** ^35^
Adams State University	3,314	HSI
Aims Community College	5,982	HSI
Arapahoe Community College	12,421	eHSI
Colorado Christian University	7,398	
Colorado College	2,118	
Colorado School of Mines	6,209	
Colorado State University—Fort Collins	33,083	
Colorado State University—Global Campus	12,381	
Community College of Aurora	8,026	HSI
Community College of Denver	8,556	HSI
Denver Seminary	908	
Emily Griffith Technical College	4,327	
Fort Lewis College	3,332	
Front Range Community College	19,259	eHSI
Iliff School of Theology	286	
Johnson & Wales University—Denver	1,170	eHSI
Metropolitan State University of Denver	20,304	HSI
Naropa University	966	
Pickens Technical College	1,011	
Pikes Peak Community College	13,275	eHSI
Red Rocks Community College	7,355	eHSI
Regis University	8,341	eHSI
United States Air Force Academy	4,276	
University of Colorado Boulder	35,338	
University of Colorado Colorado Springs	12,932	eHSI
University of Colorado Denver/Anschutz Medical Campus	24,839	eHSI
University of Denver	11,434	
University of Northern Colorado	13,399	eHSI

eHSI, emerging Hispanic-serving institution; HSI, Hispanic-serving institutions.

Of the 39 total nonprofit institutions, 11 fall outside our definition of access, accounting for ∼38,900 students, or 12.1% of Coloradan students (Tables 2 and 3; Figure 1). Of these, ∼17,800 students (45.7%) attend an HSI and 16,700 (42.9%) attend an eHSI (Table 4). Overall, 34,500 students (88.7%) attend a MSI that is over 30 minutes from an abortion provider (see Tables 4 and 5 for descriptive statistics). Six (6) of the institutions identified as lacking access are Hispanic Serving Institutions, while two (2) are Emerging Hispanic Serving Instututions.^35^

**Table 2.** Identified Institutions Without Access

**Institution**	**Total enrollment** ^27^	**Hispanic-serving status** ^35^
Colorado Mesa University	9,591	eHSI
Colorado Mountain College	5,934	eHSI
Colorado Northwestern Community College	1,201	eHSI
Colorado State University—Pueblo	6,639	HSI
Lamar Community College	811	HSI
Morgan Community College	1,383	HSI
Northeastern Junior College	1,547	
Otero Junior College	1,330	HSI
Pueblo Community College	5,991	HSI
Trinidad State Junior College	1,663	HSI
Western State Colorado University	2,814	

**Table 3.** Identified Institutions Without Access

**Institution**	**Total enrollment** ^27^	**Hispanic-serving status** ^35^
Colorado Mesa University	9,591	eHSI
Colorado Mountain College	5,934	eHSI
Colorado Northwestern Community College	1,201	eHSI
Colorado State University—Pueblo	6,639	HSI
Lamar Community College	811	HSI
Morgan Community College	1,383	HSI
Northeastern Junior College	1,547	
Otero Junior College	1,330	HSI
Pueblo Community College	5,991	HSI
Trinidad State Junior College	1,663	HSI
Western State Colorado University	2,814	

**Table 4.** Descriptive Statistics of Institutional Demographics

**Percent enrollment**	**Drive-time**	***n***	**Mean**	**Std. dev.**	**Min.**	**Max.**
Female	Overall	39	0.46	0.02	0.29	0.79
	30 Minutes	28	0.47	0.02	0.32	0.79
	45 Minutes	2	0.37	0.09	0.29	0.46
	60 Minutes	2	0.43	0.10	0.33	0.52
	No access	7	0.45	0.01	0.42	0.47
American Indian or Alaskan Native	Overall	39	0.01	0.04	0.00	0.26
	30 Minutes	28	0.02	0.05	0.00	0.26
	45 Minutes	2	0.02	0.02	0.00	0.03
	60 Minutes	2	0.01	0.00	0.01	0.01
	No access	7	0.01	0.00	0.00	0.01
Female American Indian or Alaskan Native	Overall	39	0.02	0.05	0.00	0.30
	30 Minutes	28	0.02	0.05	0.00	0.30
	45 Minutes	2	0.02	0.02	0.00	0.03
	60 Minutes	2	0.01	0.00	0.00	0.01
	No access	7	0.01	0.00	0.00	0.01
Asian	Overall	39	0.03	0.02	0.00	0.08
	30 Minutes	28	0.03	0.02	0.01	0.08
	45 Minutes	2	0.01	0.00	0.01	0.01
	60 Minutes	2	0.01	0.00	0.01	0.01
	No access	7	0.01	0.00	0.00	0.02
Female Asian	Overall	39	0.03	0.02	0.00	0.08
	30 Minutes	28	0.04	0.02	0.01	0.08
	45 Minutes	2	0.01	0.00	0.01	0.01
	60 Minutes	2	0.01	0.00	0.01	0.01
	No access	7	0.01	0.01	0.00	0.02
Black or African American	Overall	39	0.05	0.04	0.01	0.19
	30 Minutes	28	0.06	0.04	0.01	0.19
	45 Minutes	2	0.04	0.01	0.03	0.04
	60 Minutes	2	0.03	0.00	0.03	0.03
	No access	7	0.03	0.01	0.01	0.04
Female black or African American	Overall	39	0.05	0.04	0.01	0.20
	30 Minutes	28	0.06	0.05	0.01	0.20
	45 Minutes	2	0.02	0.00	0.02	0.02
	60 Minutes	2	0.02	0.00	0.02	0.02
	No access	7	0.01	0.01	0.01	0.02
Hispanic or Latino/a	Overall	39	0.18	0.10	0.04	0.44
	30 Minutes	28	0.17	0.09	0.04	0.44
	45 Minutes	2	0.24	0.12	0.15	0.32
	60 Minutes	2	0.17	0.09	0.10	0.23
	No access	7	0.22	0.11	0.10	0.42
Female Hispanic or Latino/a	Overall	39	0.19	0.11	0.04	0.49
	30 Minutes	28	0.18	0.11	0.04	0.47
	45 Minutes	2	0.23	0.13	0.14	0.32
	60 Minutes	2	0.18	0.09	0.11	0.24
	No access	7	0.24	0.13	0.12	0.49
Native Hawaiian or Pacific Islander	Overall	39	0.00	0.00	0.00	0.01
	30 Minutes	28	0.00	0.00	0.00	0.01
	45 Minutes	2	0.00	0.00	0.00	0.00
	60 Minutes	2	0.00	0.00	0.00	0.00
	No access	7	0.00	0.00	0.00	0.01
Female Native Hawaiian or Pacific Islander	Overall	39	0.00	0.00	0.00	0.01
	30 Minutes	28	0.00	0.00	0.00	0.01
	45 Minutes	2	0.00	0.00	0.00	0.00
	60 Minutes	2	0.00	0.00	0.00	0.01
	No access	7	0.00	0.00	0.00	0.01
White or Caucasian	Overall	39	0.60	0.11	0.31	0.83
	30 Minutes	28	0.60	0.12	0.31	0.83
	45 Minutes	2	0.53	0.05	0.49	0.56
	60 Minutes	2	0.66	0.03	0.64	0.68
	No access	7	0.62	0.11	0.41	0.73
Female white or Caucasian	Overall	39	0.60	0.14	0.26	0.84
	30 Minutes	28	0.59	0.14	0.26	0.84
	45 Minutes	2	0.53	0.05	0.50	0.57
	60 Minutes	2	0.65	0.02	0.64	0.66
	No access	7	0.63	0.13	0.37	0.76
Two or more	Overall	39	0.04	0.02	0.01	0.08
	30 Minutes	28	0.04	0.02	0.01	0.08
	45 Minutes	2	0.03	0.01	0.02	0.03
	60 Minutes	2	0.02	0.01	0.02	0.03
	No access	7	0.03	0.01	0.02	0.04
Female two or more	Overall	39	0.04	0.02	0.01	0.09
	30 Minutes	28	0.04	0.02	0.01	0.09
	45 Minutes	2	0.02	0.01	0.02	0.03
	60 Minutes	2	0.02	0.01	0.02	0.03
	No access	7	0.03	0.01	0.02	0.04
Unknown	Overall	39	0.06	0.05	0.00	0.28
	30 Minutes	28	0.05	0.03	0.00	0.13
	45 Minutes	2	0.14	0.19	0.01	0.28
	60 Minutes	2	0.10	0.05	0.06	0.14
	No access	7	0.07	0.04	0.02	0.12
Female unknown	Overall	39	0.06	0.05	0.00	0.31
	30 Minutes	28	0.05	0.03	0.00	0.11
	45 Minutes	2	0.16	0.22	0.01	0.31
	60 Minutes	2	0.11	0.08	0.05	0.16
	No access	7	0.06	0.03	0.01	0.11
Nonresident	Overall	39	0.03	0.03	0.00	0.11
	30 Minutes	28	0.03	0.03	0.00	0.11
	45 Minutes	2	0.00	0.01	0.00	0.01
	60 Minutes	2	0.01	0.01	0.00	0.01
	No access	7	0.02	0.02	0.00	0.05
Female nonresident	Overall	39	0.02	0.02	0.00	0.09
	30 Minutes	28	0.03	0.03	0.00	0.09
	45 Minutes	2	0.00	0.00	0.00	0.00
	60 Minutes	2	0.01	0.01	0.00	0.01
	No access	7	0.01	0.01	0.00	0.05

**Table 5.** Descriptive Statistics of Zip Code Tabulation Area Demographics

**Percent of population**	**Drive-time**	***n***	**Mean**	**Std. dev.**	**Min.**	**Max.**
Female	Overall	526	0.47	0.11	0.00	1.00
	30 Minutes	267	0.48	0.10	0.00	1.00
	45 Minutes	49	0.45	0.13	0.00	0.66
	60 Minutes	43	0.43	0.13	0.00	0.67
	No access	167	0.47	0.10	0.00	0.82
Lacking health insurance coverage	Overall	526	0.10	0.08	0.00	1.00
	30 Minutes	267	0.09	0.06	0.00	0.39
	45 Minutes	49	0.11	0.14	0.00	1.00
	60 Minutes	43	0.09	0.06	0.00	0.22
	No access	167	0.11	0.08	0.00	0.44
Female subset age 18–24	Overall	526	0.09	0.11	0.00	1.00
	30 Minutes	267	0.11	0.11	0.00	0.99
	45 Minutes	49	0.06	0.04	0.00	0.17
	60 Minutes	43	0.06	0.08	0.00	0.36
	No access	167	0.08	0.11	0.00	1.00
Female subset age 25–34	Overall	526	0.15	0.11	0.00	1.00
	30 Minutes	267	0.17	0.10	0.00	1.00
	45 Minutes	49	0.12	0.07	0.00	0.03
	60 Minutes	43	0.13	0.14	0.00	0.83
	No access	167	0.13	0.10	0.00	0.67
Female subset age 35–45	Overall	526	0.15	0.10	0.00	1.00
	30 Minutes	267	0.17	0.10	0.00	1.00
	45 Minutes	49	0.11	0.06	0.00	0.25
	60 Minutes	43	0.13	0.11	0.00	0.51
	No access	167	0.14	0.10	0.00	0.68
Female subset age 45–64	Overall	526	0.36	0.14	0.00	1.00
	30 Minutes	267	0.34	0.13	0.00	1.00
	45 Minutes	49	0.37	0.15	0.00	0.25
	60 Minutes	43	0.40	0.17	0.00	0.51
	No access	167	0.37	0.16	0.00	0.68
American Indian or Alaskan Native	Overall	526	0.01	0.05	0.00	0.94
	30 Minutes	267	0.01	0.06	0.00	0.91
	45 Minutes	49	0.01	0.01	0.00	0.05
	60 Minutes	43	0.01	0.04	0.00	0.27
	No access	167	0.01	0.03	0.00	0.35
Asian	Overall	526	0.01	0.02	0.00	0.14
	30 Minutes	267	0.02	0.03	0.00	0.14
	45 Minutes	49	0.01	0.01	0.00	0.03
	60 Minutes	43	0.00	0.00	0.00	0.03
	No access	167	0.01	0.01	0.00	0.05
Black or African American	Overall	526	0.02	0.05	0.00	0.33
	30 Minutes	267	0.03	0.06	0.00	0.33
	45 Minutes	49	0.01	0.01	0.00	0.07
	60 Minutes	43	0.02	0.05	0.00	0.23
	No access	167	0.01	0.02	0.00	0.16
Hispanic or Latino/a	Overall	526	0.17	0.17	0.00	1.00
	30 Minutes	267	0.18	0.17	0.00	0.95
	45 Minutes	49	0.14	0.17	0.00	0.88
	60 Minutes	43	0.17	0.21	0.00	0.93
	No access	167	0.15	0.16	0.00	1.00
Native Hawaiian or Pacific Islander	Overall	526	0.00	0.01	0.00	0.21
	30 Minutes	267	0.00	0.01	0.00	0.07
	45 Minutes	49	0.00	0.01	0.00	0.03
	60 Minutes	43	0.01	0.03	0.00	0.21
	No access	167	0.00	0.00	0.00	0.04
White or Caucasian	Overall	526	0.91	0.16	0.00	1.00
	30 Minutes	267	0.88	0.17	0.00	1.00
	45 Minutes	49	0.93	0.15	0.00	1.00
	60 Minutes	43	0.94	0.09	0.66	1.00
	No access	167	0.94	0.14	0.00	1.00
Other	Overall	526	0.03	0.04	0.00	0.36
	30 Minutes	267	0.03	0.04	0.00	0.21
	45 Minutes	49	0.03	0.05	0.00	0.18
	60 Minutes	43	0.03	0.05	0.00	0.30
	No access	167	0.02	0.04	0.00	0.36
Non—resident	Overall	526	0.07	0.07	0.00	1.00
	30 Minutes	267	0.08	0.06	0.00	0.39
	45 Minutes	49	0.04	0.03	0.00	0.12
	60 Minutes	43	0.06	0.08	0.00	0.39
	No access	167	0.07	0.10	0.00	1.00

Independent samples *t*-tests were used to determine if there were any statistically significant mean differences in demographics between those institutions with HSI or eHSI status and those that are not an HSI or eHSI (Table 6). Statistically significant mean differences were only found in the following demographic categories: total female enrollment [*t*(18.32) = −2.40, *p* = 0.027]; total Hispanic enrollment [*t*(37.00) = −3.04, *p* = 0.004]; female Hispanic enrollment [*t*(37.00) = −2.52, *p* = 0.016]; and total white enrollment [*t*(37.00) = 2.20, *p* = 0.034].

**Table 6.** Independent Samples *t*-Test Results for Hispanic-Serving Institutions/eHSI Status Versus No Hispanic-Serving Institutions Status

**Percent enrollment**	***T* statistic *t*-Value (df)**	**Mean difference**	***p***
Female, total	−2.40 (18.32)	0.08	0.027^a^
American Indian or Alaskan Native			
Total	0.95 (15.14)	0.01	0.355
Female	0.94 (15.15)	0.02	0.361
Asian			
Total	1.10 (37.00)	0.01	0.280
Female	1.20 (37.00)	0.01	0.238
Black or African American			
Total	−0.03 (37.00)	0.00	0.975
Female	0.54 (37.00)	0.01	0.595
Hispanic or Latino/a			
Total	−3.04 (37.00)	−0.09	0.004^a^
Female	−2.52 (37.00)	−0.08	0.016^a^
Native Hawaiian or Pacific Islander			
Total	0.31 (25.13)	0.00	0.762
Female	0.30 (37.00)	0.00	0.764
White or Caucasian			
Total	2.20 (37.00)	0.08	0.034^a^
Female	1.37 (37.00)	0.06	0.179
Two or more			
Total	0.60 (23.58)	0.00	0.555
Female	0.69 (22.70)	0.00	0.498
Unknown			
Total	−1.94 (37.00)	−0.03	0.060
Female	−1.42 (37.00)	−0.02	0.165
Non—resident			
Total	1.29 (20.41)	0.01	0.212
Female	1.38 (21.44)	0.01	0.181

^a^ Significant at 0.05.

eHSI, emerging Hispanic-serving institution.

Independent samples *t*-tests were used to determine if there were any statistically significant mean differences in demographics between those institutions with a campus health center and those without a campus health center (Table 7). Statistically significant mean differences were only found in the following demographic categories: eHSI or HSI designation [*t*(37.00) = 2.25, *p* = 0.030]; total Hispanic enrollment [*t*(24.57) = 3.03, *p* = 0.006]; male Hispanic enrollment [*t*(25.48) = 2.73, *p* = 0.011]; female Hispanic enrollment [*t*(23.72) = 3.11, *p* = 0.005]; total two or more races enrollment [*t*(37.00) = 2.20, *p* = 0.034]; female two or more races enrollment [*t*(37.00) = −2.70, *p* = 0.010]; male nonresident enrollment [*t*(27.58) = −2.34, *p* = 0.027].

**Table 7.** Independent Samples *t*-Test Results for Presence of Campus Health Center

**Percent enrollment**	***T* statistic *t*-Value (df)**	**Mean difference**	***p***
Female, total	0.37 (37.00)	0.01	0.712
American Indian or Alaskan Native			
Total	−0.67 (37.00)	−0.01	0.509
Female	−0.65 (37.00)	−0.01	0.518
Asian			
Total	−1.20 (37.00)	−0.01	0.240
Female	−1.38 (37.00)	−0.01	0.175
Black or African American			
Total	1.55 (37.00)	0.02	0.130
Female	1.40 (26.81)	0.02	0.172
Hispanic or Latino/a			
Total	3.03 (24.57)	0.08	0.006^a^
Female	3.11 (23.72)	0.10	0.005^a^
Native Hawaiian or Pacific Islander			
Total	1.47 (37.00)	0.00	0.151
Female	1.03 (37.00)	0.00	0.311
White or Caucasian			
Total	−1.17 (28.14)	−0.04	0.252
Female	−1.22 (26.86)	−0.05	0.233
Two or more			
Total	−2.44 (37.00)	−0.01	0.020^a^
Female	−2.70 (37.00)	−0.02	0.010^a^
Unknown			
Total	−0.80 (37.00)	−0.01	0.430
Female	−1.16 (37.00)	−0.02	0.253
Non—resident			
Total	−1.94 (27.83)	−0.02	0.062
Female	−1.29 (31.25)	−0.01	0.208

^a^ Significant at 0.05.

ANOVA was used to determine if there were any statistically significant mean differences in demographics between institutions with travel times of <30 minutes, 35 to 45 minutes, 45 to 60 minutes, and institutions without access.

Statistically significant mean differences were only found in the following demographic categories: total Asian enrollment [*F*(3,35) = 8.28, *p* < 0.001]; male Asian enrollment [*F*(3,35) = 7.70, *p* < 0.001]; female Asian enrollment [*F*(3,35) = 8.18, *p* < 0.001]; and female unknown enrollment [*F*(3,35) = 4.04, *p* = 0.014]. All analyses were followed-up with planned contrasts comparing (*a*) institutions without access to those with any level of access, (*b*) those with access under 45 minutes and those without access within 45 minutes, and finally (*c*) those with access in under 30 minutes to those without access in under 30 minutes.

Only the following contrasts showed statistically significant results: total Asian enrollment (*a* [*t*(13.64) = 4.05, *p* = 0.001], *b* [*t*(30.01) = 7.42, *p* < 0.001], *c* [*t*(29.43) = 8.20, *p* < 0.001]); female Asian enrollment (*a* [*t*(10.19) = 3.44, *p* = 0.006], *b* [*t*(31.78) = 6.81, *p* < 0.001], *c* [*t*(30.05) = 8.13, *p* < 0.001]; female black enrollment *a* [*t*(29.28) = 4.88, *p* < 0.001], *b* [*t*(29.51) = 4.41, *p* < 0.001], *c* [*t*(27.88) = 4.10, *p* < 0.001]; total two or more *c* [*t*(35.00) = 2.49, *p* = 0.018]; female two or more (*c* [*t*(35.00) = 2.54, *p* = 0.016]); and female nonresident (*c* [*t*(24.05) = 3.24, *p* = 0.003]).

ANOVA was used to determine if there were any statistically significant mean differences in demographics between ZTCAs with travel times of <30 minutes, 35 to 45 minutes, 45 to 60 minutes, and institutions without access (Table 8). Statistically significant mean differences were only found in the following demographic categories: female population [*F*(3,522) = 3.58, *p* = 0.014]; no health insurance coverage [*F*(3,522) = 3.65, *p* = 0.013]; females age 18 to 24 [*F*(3,522) = 6.09, *p* < 0.001]; females age 25 to 34 [*F*(3,522) = 8.42, *p* < 0.001]; females age 35 to 44 [*F*(3,522) = 7.60, *p* < 0.001]; white population [*F*(3,522) = 7.30, *p* < 0.001]; black population [*F*(3,522) = 12.75, *p* < 0.001]; Asian population [*F*(3,522) = 43.05, *p* < 0.001]; Native Hawaiian or Pacific Islander population [*F*(3,522) = 2.85, *p* = 0.037]; other race population [*F*(3,522) = 3.49, *p* = 0.016]; and nonresident population [*F*(3,522) = 4.55, *p* = 0.004].

**Table 8.** Analysis of Variance Summary Table for Institutional Drive Time Access

**Percent enrollment**	**Levene statistic (3, 35)**	**HOV *p-*value**	**SS_btwn_**	**SS_in_**	**SS_tot_**	***F*(3, 35)**	**ANOVA *p-*value**
Female, total	1.87	0.153	0.02	0.36	0.38	0.71	0.554
American Indian or Alaskan Native							
Total	0.42	0.738	0.00	0.06	0.06	0.09	0.967
Female	0.40	0.753	0.00	0.08	0.08	0.10	0.960
Asian							
Total	4.37	0.010	0.01	0.01	0.01	8.28	<0.001^a^
Female	3.64	0.022	0.01	0.01	0.01	8.18	<0.001^a^
Black or African American							
Total	2.35	0.089	0.01	0.05	0.06	1.33	0.281
Female	3.39	0.029	0.01	0.06	0.07	2.34	0.090
Hispanic or Latino/a							
Total	0.22	0.879	0.02	0.32	0.34	0.85	0.477
Female	0.17	0.919	0.02	0.43	0.45	0.56	0.646
Native Hawaiian or Pacific Islander							
Total	1.33	0.282	0.00	0.00	0.00	0.16	0.921
Female	1.79	0.166	0.00	0.00	0.00	0.39	0.758
White or Caucasian							
Total	0.71	0.555	0.02	0.48	0.50	0.48	0.701
Female	0.85	0.478	0.02	0.67	0.69	0.39	0.760
Two or more							
Total	2.25	0.100	0.00	0.01	0.01	2.45	0.080
Female	2.40	0.085	0.00	0.01	0.02	2.57	0.070
Unknown							
Total	19.56	<0.001	0.02	0.08	0.10	2.84	0.052
Female	33.11	<0.001	0.03	0.08	0.11	4.04	0.014^a^
Non—resident							
Total	2.51	0.075	0.00	0.03	0.03	0.96	0.421
Female	3.70	0.021	0.00	0.02	0.02	1.33	0.281

^a^ Significant at 0.05.

ANOVA, analysis of variance; HOV, homogeneity of variance.

All analyses were followed up with planned contrasts comparing (*a*) ZCTAs without access to those with any level of access, (*b*) those with access under 45 minutes and those without access within 45 minutes, and finally (*c*) those with access in under 30 minutes to those without access in under 30 minutes (Tables 9–11).

**Table 9.** Planned Contrast Summary Table for Institutional Drive Time Access

**Percent enrollment (drive-time in min.)**	**Mean difference**			***t*-Value (df)**			***p***		
	**>60:<60**	**>45:<45**	**>30:<30**	**>60:<60**	**>60:<60**	**>45:<45**	**>30:<30**	**>45:<45**	**>60:<60**
Female, total	0.07	0.03	−1.16	0.47 (35.00)	0.31 (35.00)	1.34 (35.00)	0.643	0.759	0.189
American Indian or Alaskan Native									
Total	0.01	0.02	0.02	0.20 (35.00)	0.37 (35.00)	0.35 (35.00)	0.841	0.711	0.730
Female	0.01	0.02	0.02	0.19 (35.00)	0.40 (35.00)	0.39 (35.00)	0.850	0.695	0.702
Asian									
Total	0.02	0.03	0.08	4.05 (8.23)	7.42 (30.01)	8.20 (29.43)	0.001^a^	<0.001^a^	<0.001^a^
Female	0.02	0.03	0.08	3.44 (10.19)	6.81 (31.78)	8.13 (30.05)	0.006^a^	<0.001^a^	<0.001^a^
Black or African American									
Total	0.04	0.04	0.08	0.64 (35.00)	0.91 (35.00)	1.65 (35.00)	0.525	0.370	0.107
Female	0.06	0.04	0.11	4.88 (29.28)	4.41 (29.51)	4.10 (27.88)	<0.001^a^	<0.001^a^	<0.001^a^
Hispanic or Latino/a									
Total	−0.09	0.02	−0.13	0.60 (35.00)	0.16 (35.00)	1.09 (35.00)	0.555	0.874	0.281
Female	−0.12	0.00	−0.10	0.70 (35.00)	0.01 (35.00)	0.76 (35.00)	0.490	0.991	0.453
Native Hawaiian or Pacific Islander									
Total	0.00	0.00	0.00	0.00 (35.00)	0.31 (35.00)	0.49 (35.00)	0.999	0.758	0.630
Female	0.00	0.00	0.00	0.36 (35.00)	1.07 (35.00)	0.37 (35.00)	0.608	0.523	0.209
White or Caucasian									
Total	−0.06	−0.15	0.00	0.36 (35.00)	1.17 (35.00)	0.01 (35.00)	0.723	0.250	0.994
Female	−0.12	−0.15	−0.01	0.58 (35.00)	1.03 (35.00)	0.23 (35.00)	0.563	0.310	0.820
Two or more									
Total	0.01	0.02	0.05	0.42 (35.00)	0.97 (35.00)	2.49 (35.00)	0.681	0.338	0.018^a^
Female	0.01	0.02	0.06	0.45 (35.00)	0.88 (35.00)	2.54 (35.00)	0.656	0.387	0.016^a^
Unknown									
Total	0.08	0.02	−0.16	0.53 (1.46)	0.17 (1.20)	−1.13 (1.24)	0.667	0.888	1.568
Female	0.14	0.04	−0.19	0.83 (1.42)	0.25 (1.29)	−1.14 (1.31)	0.524	0.839	1.579
Non—resident									
Total	−0.01	0.01	0.06	0.26 (35.00)	0.23 (35.00)	1.68 (35.00)	0.796	0.817	0.101
Female	0.00	0.01	0.06	−0.10 (8.32)	0.75 (5.27)	3.24 (24.05)	1.077	0.485	0.003^a^

^a^ Significant at 0.05.

**Table 10.** Analysis of Variance Summary Table for Zip Code Tabulation Area Drive Time Access

**Percent population**	**Levene statistic (3, 522)**	**HOV *p-*value**	**SS_btwn_**	**SS_in_**	**SS_tot_**	***F*(3, 35)**	**ANOVA *p-*value**
Female	3.74	0.011^a^	0.12	5.88	6.00	3.58	0.014^a^
Lacking health insurance coverage	1.37	0.250	0.08	11.03	11.11	3.65	0.013
Female subset age 18–24	1.08	0.359	0.07	2.23	3.30	6.09	<0.001^a^
Female subset age 25–34	1.68	0.170	0.27	5.62	5.90	8.42	<0.001^a^
Female subset age 35–45	2.29	0.077	0.23	5.16	5.39	7.60	<0.001^a^
Female subset age 45–64	2.14	0.095	0.16	10.82	10.98	2.58	0.053
American Indian or Alaskan Native	0.42	0.739	0.00	1.17	1.17	0.39	0.760
Asian	65.71	<0.001^a^	0.05	0.20	0.25	43.05	<0.001^a^
Black or African American	24.79	<0.001^a^	0.07	1.02	1.09	12.75	<0.001^a^
Hispanic or Latino/a	2.37	0.070	0.16	15.35	15.51	1.84	0.138
Native Hawaiian or Pacific Islander	9.38	<0.001^a^	0.00	0.06	0.06	2.85	0.037^a^
White or Caucasian	2.59	0.052	0.52	12.50	13.02	7.30	<0.001^a^
Other	3.09	0.027^a^	0.02	0.85	0.86	3.49	0.016^a^
Non—resident	3.54 (3, 512)	0.015^a^	0.07	2.75	2.82	4.55	0.004^a^

^a^ Significant at 0.05.

**Table 11.** Planned Contrast Summary Table for Zip Code Tabulation Area Drive Time Access

**Percent enrollment (drive-time in min.)**	**Mean difference**			***t*-Value (df)**			***p***		
	**>60:<60**	**>45:<45**	**>30:<30**	**>60:<60**	**>45:<45**	**>30:<30**	**>60:<60**	**>45:<45**	**>30:<30**
Female	−0.03	−0.03	−0.10	−0.80 (335.20)	−1.19 (113.48)	−2.94 (195.84)	1.577	1.764	1.996
Lacking health insurance coverage	−0.05	0.00	0.05	2.01 (522.00)	0.23 (522.00)	1.99 (522.00)	0.045^a^	0.817	0.047^a^
Female subset age 18–24	−0.02	−0.03	−0.13	0.74 (522.00)	1.08 (522.00)	4.27 (522.00)	0.458	0.283	<0.001^a^
Female subset age 25–34	0.03	−0.03	−0.14	1.00 (522.00)	1.34 (522.00)	4.66 (522.00)	0.319	0.182	<0.001^a^
Female subset age 35–45	0.00	−0.01	−0.13	0.06 (522.00)	0.49 (522.00)	4.57 (522.00)	0.954	0.623	<0.001^a^
Female subset age 45–64	−0.02	0.06	0.10	0.38 (522.00)	1.86 (522.00)	2.49 (522.00)	0.702	0.064	0.013^a^
American Indian or Alaskan Native	0.01	0.00	−0.01	0.69 (522.00)	0.06 (522.00)	0.54 (522.00)	0.491	0.954	0.588
Asian	0.02	−0.02	−0.06	5.06 (417.81)	−10.31 (390.41)	−11.90 (312.35)	<0.001^a^	1.8/00	1.879
Black or African American	0.04	−0.02	−0.06	3.94 (110.76)	−1.81 (74.91)	−4.94 (254.09)	<0.001^a^	1.926	2.000
Hispanic or Latino/a	0.04	−0.01	−0.10	0.74 (522.00)	0.20 (522.00)	1.95 (522.00)	0.459	0.839	0.052
Native Hawaiian or Pacific Islander	0.01	0.00	0.00	1.42 (522.00)	0.62 (522.00)	0.62 (522.00)	0.161	0.538	0.535
White or Caucasian	−0.09	0.07	0.18	1.81 (522.00)	2.06 (522.00)	3.98 (522.00)	0.071	0.040^a^	<0.001^a^
Other	0.03	−0.01	−0.02	2.15 (205.26)	−1.29 (104.98)	−1.56 (170.85)	0.033^a^	1.800	1.879
Non—resident	−0.04	0.00	−0.07	−1.45 (225.21)	0.28 (104.79)	−3.89 (210.16)	1.850	0.779	2.000

^a^ Significant at 0.05.

Only the following contrasts showed statistically significant results: no health insurance coverage (*a* [*t*(522.00) = 2.01, *p* = 0.045], *c* [*t*(522.00) = 1.99, *p* = 0.047]); females age 18 to 24 (*c* [*t*(522.00) = 4.27, *p* < 0.001]); females age 25 to 34 (*c* [*t*(522.00) = 4.66, *p* < 0.001]); females age 35 to 44 (*c* [*t*(522.00) = 4.57, *p* < 0.001]); white population (*b* [*t*(522.00) = 2.06, *p* = 0.040], *c* [*t*(522.00) = 3.98, *p* < 0.001]; black population (*a* [*t*(110.78) = 3.94, *p* < 0.001]); Asian population (*a* [*t*(417.81) = 5.06, *p* < 0.001]); and other race population (*a* [*t*(205.26) = 2.15, *p* = 0.033]).

## Discussion

Our study is the first in Colorado to look at the topic of abortion through the lens of college student access. By utilizing ANOVA to analyze ZCTA demographics, we demonstrate that there are statistically significant differences in demographics between areas within 30-minute drive-time of an abortion provider and those in 30- to 45-minutes, 45- to 60-minutes, and over 60-minutes from a provider. These patterns differed from those of campus enrollment, indicating that minority populations on collegiate campuses face different barriers to reproductive health access than the noncollegiate population.

Although this study acts as a starting point into understanding the topic of abortion access, it serves as a reminder that this topic is one where dominant discourse prevails. The combination of racial and socioeconomic status, as consistent with intersectionality, demonstrated that a significant portion of Colorado is left out of the abortion conversation.

In 2019, Progress Colorado placed a sign at state line of Utah and Colorado in protest of Utah HB0136,^36^ making abortion after 18 weeks either a second- or third-degree felony depending on which section is violated.^16^ This sign was placed along the I-70 corridor heading Eastbound into Colorado and attempts to highlight how individuals need to leave their home state to access this medical procedure.^36^ However, this billboard misrepresents the access available in Colorado. As evidenced in our analyses, the nearest clinics offering this procedure is over 200 miles driving South of this sign or over 300 miles driving East.

This is misleading to the general public, who may not realize that the Western slope, mountain region, and a large percentage of Southern Colorado remain without access to safe and legal abortion. It also creates a false narrative for those individuals seeking this procedure by implying that they are close without revealing that they still have a minimum of a 4-hour drive before reaching their destination. While the sign correctly indicates that abortion is safe and legal in Colorado, it is important to highlight and question why accessibility is left out of the larger conversation.

This conversation is of particular importance when looking at the potential impact of Texas Senate Bill 8 (SB8), which limited women in Texas's ability to obtain a safe and legal abortion within state lines after 6 weeks of pregnancy.^37^ This has resulted in a drastic increase of abortion patients across state lines, including New Mexico and Colorado, who do not require a waiting period for individuals seeking an abortion.^38^ Since the inception of SB8, Colorado has seen a 130% increase in abortion patients.^39^

Medoff^40^ found that black and Hispanic women were seeking abortion services at higher rates than their white counterparts, with Hispanic women utilizing services at over double the white rate. Despite this, our analyses indicate that the colleges lacking reasonable access to an abortion clinic are largely Emerging Hispanic Serving Institutions or full Hispanic Serving Institutions in lower income communities across the state. Our theoretical framework leads us to believe this to be because of internal bias and stereotyping of Hispanic communities, specifically Hispanic women. Hispanic women are largely portrayed as *Marianistas*, or women who are “mothers, nurturers, caregivers, and willing to serve.”^41^

There are also heterogeneous perceptions of Hispanic women having multiple children at young ages, wanting children as a way of avoiding deportation, in addition to media portrayals, which hypersexualize the Hispanic woman.^42^ The data from this study reveal a need to further examine the intersection of race/ethnicity, student services, and health care to generate a more complete picture of the ways reproductive health care is, or is not, accessible for rural Coloradans and Hispanic communities.

Introduced in 1970, Title X grant funding was created to ensure access to family planning services, contraception, supplies, and information to patients across the United States with a preference to serving low-income individuals.^43^ In Spring of 2019, the federal government introduced modifications to Title X (commonly referred to as the “gag rule”), which states that providers who offer abortion services can no longer receive Title X funds nor are providers receiving Title X funds allowed to refer patients to abortion providers.^44^

In August of 2019, Planned Parenthood pulled out of receiving Title X funding so that they could continue to provide needed services to the communities they serve.^45^ As this initial analysis was conducted shortly after the introduction of the “gag rule” we are yet to see the implications Title X modifications may have on access in Colorado where a larger percentage of abortion providers are associated with Planned Parenthood of the Rockies.

### Recommendations

We recommend that future research incorporate a qualitative approach to understand how students in these underserved communities navigate their options when unplanned pregnancies occur. Specifically, studies assessing student and rural individuals' perceptions on travel time and difficulties are needed to answer the question of what access means regarding rural health care. These studies should include addressing the difficulties lower income people may face, including unreliable transportation, a lack of public transit, and how the costs associated with travel to access health care are met.

In addition, studies assessing the practices at campus health centers are needed, as well as resources at campuses lacking dedicated health facilities. Of particular interest is the intersection of the Colorado Community College System and local public health organizations, as 8 of the 13 system institutions are without access.

As the impact of Title X changes is still unknown, we suggest a follow-up study conducted both 3 and 5 years after these changes have been put in place. This would allow a more thorough understanding of how access points change over time, especially as our analyses heavily utilizes Planned Parenthood data. In addition, these analyses need to be conducted when new legislation is put into place in nearby states. The recent restrictive regulations put into place in the State of Texas (SB8) have resulted in a larger need for Colorado abortion providers,^46^ indicating the porous nature of state boundaries in the United States.

## Abbreviations Used

ACS: American Community Survey
ANOVA: analysis of variance
COVID-19: coronavirus disease 2019
eHSI: emerging Hispanic-serving institution
GIS: Geographic Information Science
HOV: homogeneity of variance
HSI: Hispanic-serving institutions
IPEDS: Integrated Postsecondary Education Data System
MSI: Minority Serving Institution
SB8: Senate Bill 8
ZCTA: Zip Code Tabulation Area

## Notes

^1^
The term “woman” is a contested one. We prescribe to the idea that gender is flexible, nonbinary, and may change over one's life-course. However, for the purpose of this article, **we define woman as an** individual assigned the gender identity of females at birth. We are cognizant of that fact that there are individuals who do not identify within the socially constructed gender binary of male and female, but who are still capable of becoming pregnant.
